# Editor’s Book Table

**Published:** 1870-09

**Authors:** 


### Editor's Book Table.

[Note.—All works reviewed in the columns of the Chicago Medical Journal may be found in the extensive stock of W. B. Keen & Cooke, whose catalogue of Medical Books will be sent to any address upon request.]

*The Practice of Medicine.* By Thos. Hawkes Tanner, M.D., F.L.S., M.R.C.P., F.R.S.L., etc., etc. Fifth American, from the sixth London edition; thoroughly revised. Philadelphia: Lindsay & Blakiston. 1870. Pp. 1200, cloth. $6.00.

Dr. Tanner's "Practice" is very popular in England, the last edition, July, 1865, having been exhausted a considerable time before the author's large professional practice permitted him to revise and enlarge the work. It is the record of the learning and experience of an accomplished practitioner who had well illustrated the truth of Dr. Arnold's remark which he says has lessened the labor and anxiety incident to the preparation of each new edition: "That so long as you humbly learn, so long you may hopefully teach."

Not much space is given to general considerations, but his descriptions are uniformly both concise and perspicuous. He aims to give the student and busy practitioner that instruction which he needs for the immediate application of the art. Hence the therapeutic portion of the book may be said to contain its distinguishing characteristics. This is illustrated in the Appendix of Formulæ, constantly referred to in the text, which alone contains 117 pages. Under this heading, however, he refers to dietetic preparations, particular localities sought on account of their climate, and to the prominent mineral watering places.

Many practitioners object to the books that they are too indefinite in their therapeutic suggestions, but no such fault can be found with Dr. Tanner. He believes in actual medication, and although individuals may differ from him as to some of his methods and means, there is no mistaking his advice. A practitioner thoroughly grounded in the principles of medicine may, nevertheless, oftentimes be at a loss for the particular means to be used in the case before him, and here Dr. Tanner's book will about invariably afford him ideas by which both his patient and himself will profit. The sciolist will be likely to degenerate into bald routine by its easy chart. The amount of additional matter in the present edition is about 365 pages.

*A Treatise on the Theory and Practice of Obstetrics.* By WM. H. BYFORD, A.M., M.D., Professor of Obstetrics and Diseases of Women and Children in the Chicago Medical College, etc., Author of the Practice of Medicine and Surgery applied to the Diseases and Accidents incident to Women, Chronic Inflammation of the Unimpregnated Uterus, etc. New York: William Wood & Co., 61 Walker street. 1870. Pp. 457. $4.50.

The late period in the month at which this treatise was received prevents extended notice. Prof. Byford has been long and favorably known to the professional public by his numerous communications to the medical press, his previously published elaborate books, and by his widely extended private and consultation practice.

Long and varied experience, scholarly habits of study, and excellent original capacity, have enabled him to lay before the profession contributions to the general stock of knowledge which deserve and receive the meed of high favor. He has achieved enviable success among medical writers.

The present volume is thoroughly practical in its character, with numerous woodcut illustrations well adapted to explain the text. Not much space is devoted to the discussion of mooted points, the author usually dismissing them with a simple announcement of his own opinion. We notice that he mentions favorably in cases where mere extraction is sought, Prof. Evans' Obstetric Extractor. Taken as a whole, although obliged to dissent from some of his views on certain points, we take pleasure in commending Prof. Byford's book as fully up to the times and a successful exposition of the subject.

*On Microscopical Manipulation; being the Subject-matter of a Course of Lectures delivered before the Quekett Microscopical Club, January—April*, 1869. By W. T. SUFFOLK, F.R.M.S. Illustrated with forty-nine engravings and seven lithographs. Philadelphia: J. B. Lippincott & Co. 1870. $2.00.

A capital little book, precisely what is needed by amateur microscopists. It is briefer and more compendious than that of Beale, and we have no doubt will become highly popular as a hand-book. It contains chapters on Construction of the Microscope; Mechanical Processes; Mounting Objects Dry and in Balsam; Mounting Objects in Fluid; Illuminating Apparatus; Polarized Light; Drawing and Micrometry; an Appendix on Apparatus, with concluding notes on practical points. The illustrations are excellent.

